# A test of cognitive mediation in a 12-month physical activity workplace intervention: does it explain behaviour change in women?

**DOI:** 10.1186/1479-5868-7-32

**Published:** 2010-05-03

**Authors:** Ronald C Plotnikoff, Michael A Pickering, Ryan E Rhodes, Kerry S Courneya, John C Spence

**Affiliations:** 1 School of Education, University of Newcastle, Callaghan, NSW, Australia; 2 Faculty of Physical Education and Recreation, University of Alberta, Edmonton, AB, Canada; 3 Alberta Centre for Active Living, University of Alberta, Edmonton, AB, Canada; 4 Centre for Health Promotion Studies, School of Public Health, University of Alberta, Edmonton, AB, Canada; 5 Behavioural Medicine Laboratory, School of Exercise Science, University of Victoria, Victoria, BC, Canada

## Abstract

**Background:**

Attempts to demonstrate the efficacy of interventions aimed at increasing physical activity (PA) have been mixed. Further, studies are seldom designed in a manner that facilitates the understanding of how or why a treatment is effective or ineffective and PA intervention designs should be guided by a heavier reliance upon behavioral theory. The use of a mediating variable framework offers a systematic methodological approach to testing the role of theory, and could also identify the effectiveness of specific intervention components. The primary purpose of this paper was to test the mediating role that cognitive constructs may have played in regards to the positive effect that a workplace behavioral intervention had on leisure-time PA for women. A subsidiary purpose was to examine the cross-sectional relationships of these cognitive constructs with PA behavior.

**Methods:**

The Physical Activity Workplace Study was a randomized controlled trial which compared the effects of stage-matched and standard print materials upon self-reported leisure-time PA, within a workplace sample at 6 and 12-months. In this secondary analysis we examined the mediation effects of 14 psychosocial constructs across 3 major social-cognitive theories which were operationalized for the intervention materials and measured at baseline, 6 and 12-months. We examined change in PA and change in the psychological constructs employing a mediation strategy proposed by Baron and Kenny for: (1) the first 6-months (i.e., initial change), (2) the second 6-months (i.e., delayed change), and (3) the entire 12-months (overall change) of the study on 323 women (n = 213 control/standard materials group; n = 110 stage-matched materials group).

**Results:**

Of the 14 constructs and 42 tests (including initial, delayed and overall change) two positive results were identified (i.e., overall change in pros, initial change in experiential powerful intervention approaches processes), with very small effect sizes. However, these mediating results were eliminated after adjusting for the multiple statistical tests.

**Conclusions:**

The intervention did not change these mediators in any substantive way, and show a similar pattern to prior research where interventions generally do not result in a change in mediation of behavior change. It is important to report mediation results in randomized controlled trials whether the findings are null or positive. Future studies may wish to focus on more detailed dose-response issues between mediators and behavior, the inclusion of moderators that could affect individual change, or different mediator constructs at higher levels of measurement specificity. Continued work on innovative and more powerful PA intervention approaches are needed.

## Background

In light of the compelling evidence supporting positive effects of regular physical activity (PA) [1], a significant proportion of our population remain sedentary [2]. It is not surprising, therefore, that health researchers have turned attention to identifying and validating methods of increasing PA levels of individuals to improve the health of our population [3]. To date, attempts to demonstrate the efficacy of interventions aimed at increasing PA have been mixed. In a systematic review of 23 physical activity intervention studies, Baranowski and colleagues [4] reported that many published intervention studies had little or no impact on PA behavior. The authors also concluded that PA interventions often demonstrated effects only for a subset of targeted outcome measures or only for some subgroups at some times, but not other time points. A more recent comprehensive meta-analysis across all contexts of physical activity interventions revealed an overall small effect size (i.e., d = .31) [5]. In a meta-analysis specifically focused on workplace PA interventions, Dishman et al. [6] reported an overall small effect size (r = 0.11) across 26 studies.

According to Baranowski et al. [4], studies are seldom designed in a manner that facilitates the understanding of how or why a treatment is effective, and these authors recommend that PA intervention designs be guided by a heavier reliance upon behavioral theory. Other researchers [7,8] have made similar recommendations.

In addition to suggesting a stronger theoretical foundation, Baranowski et al. [4] also point out that the use of a mediating variable framework offers a systematic methodological approach to testing the role of theory, and could also identify the effectiveness of specific intervention components. Other researchers have made calls for an increased emphasis and utilization of mediation designs in physical activity research [9-11]. The rationale underlying this emphasis is that theory-based intervention research postulates a particular treatment will increase PA by influencing specific constructs that relate to behavior change [8]. Thus, a mediating variable framework provides a basis for examining both the overall efficacy of the intervention, and its proposed mechanism of operation [3,12].

Experimental designs that assess the relationship between *change *in cognitive mediators and *change *in behavior [4] are further enhanced when more than two assessments are included so that the time precedence requirement of causal inference can also be established [3,8]. It is important to assess mediators alongside intervention outcomes to obtain a better understanding of intervention efforts. Our current understanding of theories is predominantly based on correlational designs [13-15] and more studies need to be conducted to examine the internal validity of these theories.

The Physical Activity Workplace Study (PAWS) [16] incorporated a randomized controlled trial design to compare the effects of introducing stage-matched and standard print materials upon self-reported leisure-time PA, within a workplace sample. In addition to assessing self-reported PA prior to the intervention, and at two follow-up time points (i.e., at six and 12 months), PAWS participants also reported levels of 14 cognitive constructs at all three assessments. The selected cognitions were based on social-cognitive theories namely, Transtheoretical Model [17], Theory of Planned Behavior [18], Protection Motivation Theory [19] and Social Cognitive Theory [20] which are frequently employed in physical activity literature and were operationalized in an integrated theoretical manner for promoting behavior stage of change.

Plotnikoff et al. [16] reported a significant positive effect of the stage-matched intervention upon leisure time PA for females but not for males. However, the original PAWS report did not explore the effect of the intervention on the cognitive constructs, nor did it examine the relationships between the cognitions and leisure time PA.

Therefore, the primary purpose of these follow-up analyses was to investigate the potential mediating role that the cognitive constructs may have played in regards to the positive effect that the PAWS intervention had on leisure-time PA for women. A secondary purpose was to confirm that the theoretical constructs examined as potential mediators of PA were empirically reasonable choices, regardless of observed intervention effects. Specifically, to meet our first purpose we: (a) identified what particular cognitions changed in women, in response to the intervention, (b) determined what cognitive changes were related to PA change, and (c) evaluated whether inclusion of cognitive change within a mediation framework significantly attenuated the observed direct effect of the intervention on PA. To address our second purpose we examined bivariate and partial correlations of each cognition with PA behavior.

## Methods

### Design

A detailed description of the methods used in the PAWS study has been reported elsewhere [16,21]. In brief, the study was conducted to compare the efficacy of (1) a stage-matched intervention (based on print materials matched for PA stage of readiness), with (2) a standard print-based intervention [the Canadian Physical Activity Guide (CPAG)] [22], and (3) a control group. The primary outcome variable was self-reported leisure time PA. Participants were recruited from three large Canadian worksites via flyers, posters and e-mail notification. Each worksite was further subdivided into geographically-based sub-sites, which were then randomly assigned to one of the three study arms. A University Research Ethics Committee approved all study procedures.

In the original PAWS report the intervention was effective for females in the stage-matched treatment group, who reported greater increases in PA than women in either the standard treatment group (i.e., those who received the CPAG) or the control group. Women participants in the standard treatment and control groups did not differ in PA change [16]. According to Cohen's guidelines, there was more than adequate power (> .80; alpha < .05) with the group N's to detect a medium effect size (i.e., a mean difference of 0.5 of a standard deviation) between the three study groups (as well as between the combined standard and control group versus the stage-matched group) in the subsample of women on PA behavior over the course of the study [23]. Subsequent analyses further revealed almost no differences between the standard versus control groups on cognitive change over the duration of the study. Of the 14 social cognitive variables examined in the study, only one construct (i.e., 'perceived behavioral control') changed significantly between the groups over time (p = .03); the magnitude of the difference was small (approx .20 on the 5-point scale). In the current analyses we combined women in the standard treatment group with the control group women, and compared this new group against women who received the stage-matched print materials.

### Participants

Participants were employees from three large organizations in the province of Alberta, Canada [16]. Though 897 participants were recruited in the original study, 507 (56.5%) completed the questionnaire at all three time periods. The current analyses are restricted to women who completed the questionnaires at all three assessments, and who included sufficient information for change in PA scores to be computed (n = 326 of 661 females, 49.3%). (For consistency, it was important to employ the same N of women as reported in the primary paper [16] for our mediation analyses; therefore we did not impute the last observation carried forward technique for those missing the 6 and 12-month assessments.) Of these, three women reported changes in MET-minutes scores from Time 1 to Time 3 of greater than 5000 MET-minutes, which was substantially more than three standard deviations from the mean MET-minutes change score. Thus, these three cases were considered outliers, and were removed from subsequent analyses. The women remaining in the sample (n = 324) exhibited a mean age of 42.6 years (SD = 8.8), and were highly educated (82.0% reported having at least a college degree). Approximately three-quarters (n = 244) of the remaining sample reported being in a committed relationship (i.e., either married or common law). Two-thirds (n = 217) reported working full time, 31.5% (n = 102) worked part-time, and less than 1% of the respondents were volunteers (n = 3). Two women in the sample did not report their employment status. For this study analyses, the combined control and standard materials group consisted of 213 women, while the stage-matched group comprised 110 women; there were no statistical differences between the groups on the above-mentioned demographic characteristics.

### Measures

Questionnaires that included demographic information, self-reported leisure time PA, and several psychosocial constructs, were collected at three times: baseline, six-months, and 12-months.

#### demographic characteristics

Age, gender, marital status, education level, gross family income, employment status, height and weight were collected as part of the baseline assessment.

#### physical activity

Respondents reported frequency, duration, and level of intensity (i.e., light, moderate or strenuous) of weekly PA sessions, based on a modified version [16] of the Godin Leisure-Time Exercise Questionnaire (GLTEQ) [24]. Frequency and duration of moderate and strenuous sessions were multiplied together, then weighted by estimates of metabolic equivalents (i.e., 4.0 MET for moderate activity and 7.5 MET for strenuous activity) [25]. MET.minutes scores for each session were totalled, providing an estimate of total weekly PA. Light activity was not included in the PA calculations.

#### psychological constructs

A total of 14 psychosocial constructs from the Theory of Planned Behavior [18], Transtheoretical Model [17], Protection Motivation Theory [19], and Social Cognitive Theory [20,26], were considered as potential mediators of the intervention effect upon self-reported PA (see Plotnikoff et al. [16] for details of our rationale). Table 1 displays (a) an example item from each scale, (b) response scale anchors and number of items (c) instrument development source, and (d) reliability coefficients for each assessment in this study. Constructs scores were computed as the mean value of responses to each scale's individual items.

**Table 1:** Example items from each psychological construct scale, number of items and response scale, instrument development source(s), and reliability coefficients computed at each assessment point in this study.

Psychological construct	Sample item	Response scale (number of items)	Construct source(s) [reference number]	α_T1_ α_T2_ α_T3_
self-efficacy	*In the next six months, I am confident that I can participate in regular physical activity:* When I have to do it by myself.	"Not confident at all" to "Extremely confident" (9 items)	[43,44]	.93 .93 .94
decisional balance pros	*Over the next 6 months:* Physical activity would help reduce tension or manage stress.	"Not at all" to "Very much" (5 items)	[43,44]	.85 .83 .85
decisional balance cons	*Over the next 6 months:* Physical activity would take too much time.	"Not at all" to "Very much" (6 items)	[43,44]	.77 .75 .78
behavioral processes	*How often in the past month:* Did you set physical activity goals for yourself that you could reach?	"Never" to "Very often" (9 items)	[44]	.86 .84 .87
experiential processes	*How often in the past month:* Did warnings about health problems cause concern?	"Never" to "Very often" (10 items)	[44]	.81 .80 .82
severity	*I feel:* For me, being physically inactive would be a very bad thing.	"Definitely not" to "Definitely yes" (3 items)	[45,46]	.61 .60 .81
vulnerability	*I feel:* If I don't get enough physically activity, I would be at risk for serious health problems.	"Definitely not" to "Definitely yes" (3 items)	[45,46]	.84 .92 .91
fear	*I feel:* Not getting enough physical activity would frighten me because of the possibility of developing serious health problems.	"Definitely not" to "Definitely yes" (3 items)	[45,46]	.93 .94 .95
response efficacy	*I feel:* For me, physical activity will keep me healthy.	"Definitely not" to "Definitely yes" (3 items)	[45,46]	.83 .78 .91
attitude	*For me regular physical activity over the next 6 months will be:* "Quite Useful" to "Quite Useless"	Response scale differed for each item (i.e., enjoyable, useful, wise, interesting, relaxing, beneficial) (6 items)	[47,48]	.78 .82 .80
injunctive norms	*Over the next 6 months:* Most people in my social network want me to do regular physical activity.	"Strongly disagree" to "Strongly agree" (4 items)	[47,48]	.67 .73 .73
descriptive norms	*Over the next 6 months:* Most of my family members will participate in regular physical activity.	"Strongly disagree" to "Strongly agree" (4 items)	[47,48]	.55 .61 .63
social support	*Over the next 6 months:* People in my social network are likely to help me participate in regular physical activity.	"Strongly disagree" to "Strongly agree" (3 items)	[47,48]	.76 .73 .73
perceived behavioral control	*Over the next 6 months:* If I were really motivated participating in regular physical activity would be easy.	Response scale differed for each item (4 items)	[47]	.57 .60 .60

### Statistical Analyses

**Figure 1:**
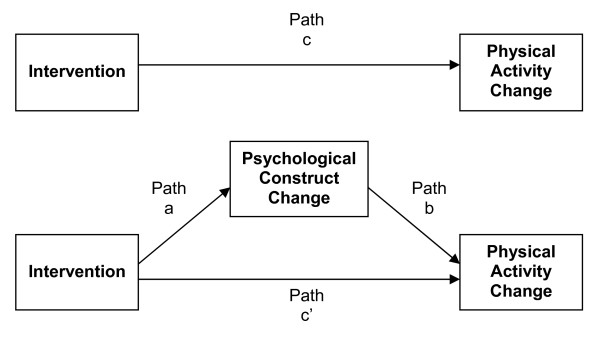
**Overview of mediation process and analysis procedure**.

Our analytical approach consisted of product of coefficients tests of mediation [27] and was guided by the previously reported findings of the PAWS study [16]. To explore the potential mediation processes, we employed a mediation strategy proposed by Baron and Kenny [28]. We first confirmed the overall stage-matched intervention effect for women (i.e., path 'c' in Figure 1) by regressing self-reported PA change upon a dummy-coded indicator of group membership (i.e., stage-matched materials vs. CPAG print materials or control group). Next, we examined the effect of the stage-matched intervention on the various psychological constructs (i.e., path 'a' in Figure 1) by separately regressing change in each of the cognitions upon intervention group membership. If the regression coefficients for both paths 'a' and 'c' were significant, we then regressed the change in PA upon the dummy-coded intervention variable and the change in the potential mediator simultaneously. This third step determined: (a) if change in the potential mediating psychological construct had a significant relationship with the change in PA, after controlling for the effect the intervention had on both (i.e., path 'b' in Figure 1), and (b) if the direct effect of the intervention upon PA change was attenuated (as compared to the direct effect when the mediator was not included in the model), as is required to infer that at least partial mediation was plausible [29]. Testing the statistical significance of the indirect effect through paths 'a' and 'b' is equivalent to that of the attenuation of path 'c' [29]. Statistical significance of the indirect effect was based upon the Sobel test statistic [30], which has been shown to be stable for single mediator models with sample sizes greater than N = 50 [31]. Based on the sample sizes of our two study groups, we had power at the 0.8 level to detect medium effect sizes for any potential mediation effects of the psychosocial constructs [32].

Although mediation analysis typically stops if either path 'c' or path 'a' is not statistically significant, in order to address our second study aim we proceeded to calculate bivariate and partial correlations (controlling for the intervention effect), between change in each of the psychological constructs and change in PA. This served as an empirical post-hoc confirmation of the appropriateness of the theory-based cognitions that were chosen as potential mediators. In other words, did changes in these cognitions relate to change in physical activity, as theory would suggest, irrespective of the effects or non-effects of the intervention.

Finally, change in PA across the entire 12-month intervention was presented in the original PAWS report [16]. However, specific examination of the pattern of change within the 12 months was not reported. In these follow-up analyses we examined change in PA and change in the psychological constructs for: (1) the first six months (i.e., initial change), (2) the second six months (i.e., delayed change), and (3) the entire 12 months (overall change) of the study. Throughout this report, references to initial change of PA and cognitions at six months (Time 2) and overall change at 12 months (Time 3) refer to residual change; post-test values were regressed onto their baseline values to create residualized change scores.

## Results

### Preliminary Analyses

Prior to performing mediation analyses all 14 cognitive constructs and leisure time PA levels were compared between study participants who provided sufficient data for these analyses (i.e., completed all three assessments and provided PA data) and those who did not (i.e., either did not complete all three assessments or did not provide sufficient data to calculate change in PA levels). Independent t-tests indicated that no significant differences on PA levels, or on 13 of the 14 psychological constructs (i.e., all p-values > .10). For the one significant construct (i.e., severity), participants who were not included in these analyses (n = 333) reported slightly lower levels (M = 4.62, SD = 0.60) than did participants who were [(n = 325; M = 4.73, SD = 0.46), t(656) = -2.60, p = .01], however the magnitude of this difference was small. Thus, although the current mediation analyses are based on approximately one-half of the participants originally recruited, there were no substantive differences in baseline characteristics between those who provided sufficient data for these analyses and those who either dropped out of the study or provided insufficient data, in regards to leisure time physical activity and the psychological constructs under consideration. Moreover, there were no drop-out differences between the study groups.

### Path c: Direct Intervention Effect on Changes in Physical Activity

The top panel of Table 2 indicates that the dummy-coded intervention group variable contributed significantly to prediction of both delayed and overall change in leisure time PA. Treatment group membership did not predict change in PA during the first six months of the study. Specifically, after controlling for individual activity levels reported at the beginning of each time period, women in the stage-matched group reported significantly higher weekly leisure time PA, by approximately 320 and 270 MET-minutes, during the second six months and over the entire 12 months respectively, as compared to women in the combined CPAG and control group. Group membership accounted for 2.6% of the variance in delayed PA change and 2.1% in overall PA change.

**Table 2:** Group means (i.e., stage-matched vs. CPAG + control), unstandardized regression coefficients, p-values, effect-size, and power for self-reported physical activity and eight of 14 potential mediating psychological constructs.

	Group Means					
	Stage-matched	Control + CPAG	B	p	R^2^	power
Physical activity						
Initial Change	-76.510	-15.640	-60.867	0.564	0.001	0.089
Delayed Change	104.121	-214.392	318.513	**0.004**	0.026	0.818
Overall Change	70.496	-198.624	269.120	**0.009**	0.021	0.746

Self-efficacy						
Initial Change	0.068	-0.015	0.083	0.207	0.005	0.243
Delayed Change	0.036	0.035	0.002	0.979	0.000	0.050
Overall Change	0.071	0.011	0.060	0.392	0.002	0.137
Pros						
Initial Change	0.093	0.001	0.092	0.216	0.005	0.235
Delayed Change	0.075	0.005	0.071	0.305	0.003	0.176
Overall Change	0.121	-0.023	0.145	**0.041**	0.013	0.533
Cons						
Initial Change	0.014	0.001	0.013	0.827	0.000	0.055
Delayed Change	-0.024	-0.011	-0.013	0.823	0.000	0.056
Overall Change	-0.014	0.008	-0.022	0.728	0.000	0.064
Experiential processes						
Initial Change	0.125	-0.023	0.148	**0.011**	0.020	0.726
Delayed Change	-0.022	0.030	-0.052	0.380	0.002	0.142
Overall Change	0.061	0.006	0.055	0.390	0.002	0.138
Behavioral processes						
Initial Change	0.036	0.037	-0.001	0.987	0.000	0.050
Delayed Change	0.076	-0.002	0.078	0.264	0.004	0.201
Overall Change	0.033	-0.015	0.048	0.491	0.001	0.106
Severity						
Initial Change	0.037	-0.012	0.049	0.356	0.003	0.152
Delayed Change	-0.028	0.000	-0.028	0.679	0.001	0.070
Overall Change	-0.027	-0.032	0.005	0.942	0.000	0.051
Vulnerability						
Initial Change	0.049	-0.081	0.131	0.152	0.006	0.298
Delayed Change	-0.003	0.009	-0.012	0.894	0.000	0.052
Overall Change	0.009	-0.029	0.037	0.664	0.001	0.072
Fear						
Initial Change	0.001	-0.011	0.012	0.910	0.000	0.051
Delayed Change	0.064	-0.047	0.111	0.213	0.005	0.238
Overall Change	0.052	-0.056	0.108	0.235	0.004	0.220

Bolded values = p < .05

### Path a: Intervention Effect on Changes in Psychological Constructs

Tables 2 and 3 display residual change score means and associated unstandardized regression coefficients, p-values, effect-sizes and power for the intervention effect upon all 14 psychological construct change scores, including initial, delayed, and overall change. Out of 42 models exploring the possible mediating role of psychological constructs, only two indicated that the stage-matched intervention contributed significantly toward predicting changes in the cognitions. On average, overall change in pros, and initial change in experiential processes, both increased approximately 0.15 units more (on a 5 point scale) for the stage-matched intervention group than for the CPAG plus control group. The intervention accounted for 1.3% and 2.0% of the variance in overall change in pros and initial change in experiential processes, respectively. Instead of adjusting p-values for the number of tests, we have reported exact p-values, effect sizes, and power estimates for all analyses in Tables 2 and 3, so that readers may evaluate significance of results for themselves. In light of effect size and power considerations, our mediation analyses were guided by uncorrected p-criteria of .05 as representing individual effects unlikely to have been observed in our samples, if in fact, no such effects would be evident in populations with similar characteristics.

**Table 3:** Group means (i.e., stage-matched vs. CPAG + control), unstandardized regression coefficients, p-values, effect-size, and power for the remaining six of 14 potential mediating psychological constructs.

	Group Means						
	Stage-matched	Control + CPAG	B	p	R^2^	power	
Response efficacy							
Initial Change	0.056	-0.007	0.063	0.187	0.005	0.261	
Delayed Change	0.046	-0.011	0.057	0.382	0.002	0.141	
Overall Change	0.073	-0.019	0.092	0.160	0.006	0.290	
Attitude							
Initial Change	-0.090	-0.003	-0.087	0.079	0.010	0.420	
Delayed Change	-0.016	0.008	-0.025	0.567	0.001	0.088	
Overall Change	-0.055	0.010	-0.066	0.187	0.005	0.261	
Injunctive norms							
Initial Change	0.080	0.054	0.026	0.640	0.001	0.075	
Delayed Change	0.024	-0.018	0.041	0.469	0.002	0.112	
Overall Change	0.062	0.001	0.061	0.288	0.004	0.186	
Descriptive norms							
Initial Change	0.056	0.009	0.046	0.507	0.001	0.101	
Delayed Change	0.054	0.005	0.049	0.466	0.002	0.113	
Overall Change	0.086	0.000	0.086	0.236	0.004	0.220	
Social support							
Initial Change	0.098	0.041	0.057	0.435	0.002	0.122	
Delayed Change	-0.017	-0.005	-0.012	0.879	< .001	0.053	
Overall Change	0.057	0.008	0.050	0.552	0.001	0.091	
Perceived behavioral control							
Initial Change	0.008	0.026	-0.018	0.798	< 0.001	0.058	
Delayed Change	0.038	-0.009	0.046	0.450	0.002	0.117	
Overall Change	0.026	-0.012	0.038	0.569	0.001	0.088	

### Path b: Effect of Cognitive Changes on Changes in Physical Activity

The pros construct showed the only significant *overall *change in response to the stage-matched intervention. Therefore, it was considered as a potential mediator only for *overall *change in leisure time PA. The path b mediation coefficient showed a significant relationship between the overall change in pros and the overall change in leisure time PA. Specifically, for every one unit of overall change in pros, weekly leisure time PA increased by 162.0 METs, after controlling for initial level of pros and the effect of the intervention.

The experiential process construct showed significant initial change, and was therefore considered a potential mediator for both the delayed and overall change in leisure time PA. However, neither the path b coefficient from initial change in experiential processes to delayed change in physical activity, nor the path b coefficient from initial change in experiential processes to overall change in PA, was statistically significant (B_delayed PA _= 58.3, p = .584; B_overall PA _= 62.4, p = .532).

### Mediation Effects (Attenuation of Path c)

Evaluating the significance of the magnitude of a mediating effect (attenuation of path c) is typically pursued only if both path a, and path b are significant. Based on our criteria, only the potential indirect effect of the stage-matched intervention upon overall change in leisure time PA, through the overall change in pros, exhibited significance of both paths, and thus, was the only mediating effect examined for statistical significance.

#### mediation via change in pros

The change in R^2 ^values when the intervention was added as a second predictor of PA change (in addition to change in pros) was .017. This R^2 ^change value was slightly smaller in magnitude than the R^2 ^value of .021 that was observed when the stage-matched intervention was the only predictor of change in leisure time PA.

The magnitude of this indirect effect was 23.50 MET-minutes (i.e., the mediating effect accounted for a 23.5 MET-minutes greater change in PA for the intervention group than for the control group), and the associated Sobel z = 1.430 was not statistically significant (p = .153). Thus, although the amount of unique variance shared between the intervention and PA was less when the change in pros was also included as a predictor than when the intervention predicted PA alone, the mediating effect was not large enough to consider change in perceived benefits of PA (pros) as a significant mediator of the intervention effect upon the self-reported changes in leisure time PA.

### Bivariate and partial correlations of cognitions with physical activity change

Tables 4 and 5 presents the bivariate and partial correlations of the cognitive changes with PA change (regardless of intervention effects on cognitions).

**Table 4:** Bivariate and partial correlations between changes in potential mediating psychological constructs and change in self-reported physical activity (females) for self-reported physical activity and nine of 14 potential mediating psychological constructs.

	Bivariate Correlations			Partial Correlations		
	Initial Change PA	Delayed Change PA	Overall Change PA	Initial Change PA	Delayed Change PA	Overall Change PA
Self-efficacy						
Initial Change	**.152**	**.199**	**.227**	**.155**	**.191**	**.220**
Delayed Change	.017	**.157**	**.153**	.016	**.166**	**.159**
Overall Change	.100	**.261**	**.291**	.101	**.261**	**.291**
Pros						
Initial Change	**.130**	.051	.093	**.133**	.036	.082
Delayed Change	-.013	**.120**	.092	-.012	**.114**	.086
Overall Change	.044	**.113**	.110	.047	.095	.096
Cons						
Initial Change	-.010	**-.124**	-.104	-.010	**-.128**	-.107
Delayed Change	.059	**-.197**	-.127	.058	**-.198**	**-.126**
Overall Change	.069	**-.233**	**-.157**	.069	**-.235**	**-.157**
Experiential processes						
Initial Change	.051	.032	.061	.055	.012	.046
Delayed Change	.067	**.165**	**.172**	.066	**.176**	**.181**
Overall Change	.077	**.164**	**.186**	.078	**.161**	**.184**
Behavioral processes						
Initial Change	**.327**	.019	**.132**	**.327**	.022	**.135**
Delayed Change	-.064	**.373**	**.310**	-.062	**.369**	**.304**
Overall Change	**.140**	**.328**	**.351**	**.141**	**.326**	**.349**
Severity						
Initial Change	**.140**	.107	**.145**	**.142**	.096	**.137**
Delayed Change	-.001	**.133**	**.125**	-.001	**.136**	**.127**
Overall Change	.055	**.162**	**.173**	.056	**.158**	**.170**
Vulnerability						
Initial Change	-.064	.087	.096	-.062	.075	.087
Delayed Change	.017	.038	.048	.017	.038	.049
Overall Change	-.015	.077	.097	-.014	.073	.094
Fear						
Initial Change	-.041	.025	-.019	-.040	.019	-.024
Delayed Change	.029	.043	.054	.031	.031	.045
Overall Change	-.012	.047	.025	-.010	.032	.014
Response efficacy						
Initial Change	.094	.032	.056	.097	.017	.045
Delayed Change	.039	**.125**	**.112**	.040	**.122**	**.109**
Overall Change	.056	**.117**	**.109**	.058	.109	.102

Bolded values = p < .05

**Table 5:** Bivariate and partial correlations between changes in potential mediating psychological constructs and change in self-reported physical activity (females) for self-reported physical activity and the remaining five of 14 potential mediating psychological constructs.

	Bivariate Correlations			Partial Correlations		
	Initial Change PA	Delayed Change PA	Overall Change PA	Initial Change PA	Delayed Change PA	Overall Change PA
Attitude						
Initial Change	-.033	**-.130**	-.090	-.036	**-.113**	-.076
Delayed Change	-.061	-.034	-.065	-.061	-.032	-.063
Overall Change	-.059	**-.121**	**-.123**	-.062	**-.110**	**-.115**
Injunctive norms						
Initial Change	.105	-.030	.003	.104	-.026	.006
Delayed Change	-.102	**.185**	**.118**	-.101	**.180**	**.113**
Overall Change	-.059	**.166**	**.112**	-.058	**.162**	.108
Descriptive norms						
Initial Change	.065	-.011	-.019	.066	-.018	-.025
Delayed Change	-.038	**.124**	.068	-.038	**.120**	.063
Overall Change	-.020	.099	.036	-.018	.088	.027
Social support						
Initial Change	.024	.054	.035	.025	.050	.031
Delayed Change	**.236**	.074	**.131**	**.237**	.072	**.130**
Overall Change	**.209**	.103	**.138**	**.211**	.094	**.132**
Perceived behavior control						
Initial Change	**.157**	.126	.055	**.157**	.030	.058
Delayed Change	.045	**.204**	**.188**	.046	**.201**	**.185**
Overall Change	.102	**.092**	**.102**	.102	**.094**	**.104**

Bolded values = p < .05

## Discussion

The primary purpose of this study was to examine the potential mediators of behavior change in a worksite-distributed PA intervention [16]. Potential mediators included constructs of the Theory of Planned Behavior, Protection Motivation Theory, the Transtheoretical Model, and Social Cognitive Theory allowing for a broad assortment of social-cognitive constructs. The results suggest a general failure of these constructs to account for mediation, and several potential reasons for this null effect are discussed below. We believe the results of this study will inform future research efforts.

Of the 14 constructs and 42 tests (including initial change, delayed change, and overall change) only two positive results were identified (i.e., overall change in pros, initial change in experiential processes), and these were of very small effect size [23]. Adjusting the alpha to account for the multiple tests, eliminate any mediation effect in this study.

Thus, it is clear that the intervention did not change these mediators in any substantive way. Low fidelity observed in our current interventions has also been found among many other trials (see Lewis et al. [8], Lubans et al, [33], and Rhodes and Pfaeffi [34] for reviews). In a recent study [35] somewhat similar to ours also reported that a lifestyle physical activity intervention in women effectively increased physical activity, but none of the proposed psychosocial constructs showed a mediating effect. In a recent review of 28 primary prevention PA trials, Rhodes and Pfaeffli [34] found that over half of the trials failed to change the proposed mediators of the intervention.

Clearly, continued innovation to increase the power of interventions is needed to bring about change in social-cognitive variables. This problem poses less of a challenge to current theories as it does to interventions; nevertheless, it does suggest that social-cognitive constructs may be difficult to change following interventions. Interestingly, in our study, several of the changes in social-cognitive constructs were related to subsequent changes in PA independent of the intervention arm. Thus, there was some evidence of validity for these mediators despite the intervention's failure to change these constructs. However, there are practical limitations to changing these constructs that also need consideration. For example, many of the constructs demonstrated very high mean values [16]. This suggests a ceiling/threshold for the possibility of change. Fishbein et al. [7] highlight the importance of demonstrating 'room for change' in psychological constructs as an important step in interventions. There are also other potential methodological artefacts such as measurement times used (too long or too short to capture changes), and attenuation of measurement error. Another explanation for the observed small changes may be explained by *response shift theory *[36]. Response shift theory refers to a change in one's self-evaluation and/or internal standard (e.g., attitude, perceived control) that result from a change in a measured variable (e.g., PA behavior). An individual for example, may have high perceptions of control before engaging in PA. However, when the individual initiates the behavior, perceptions of control may diminish as barriers to PA and other related constraints may increasingly inhibit the ability to perform PA. Although to date *response shift theory *has not been evaluated in the social-cognitive domain, this theory may provide one explanation for the observed positive behavioral changes (i.e., PA) while cognitive variables remained relatively stable.

In our study, there was an intervention effect on behavior, despite the intervention's general inability to change the examined psychological mediators. A failure to show mediation by social-cognitive variables despite behavior changes, is common [8,36-39]; indeed most intervention effects on behavior have not been completely accounted for by the proposed mediators [8,34]. Further, a recent study [40] that reviewed mediators of dietary behavior change observed that interventions were relatively unsuccessful in changing mediators. The most powerful and consistent mediator of behavior change interventions in past research has been the behavioral processes of change [8,34], however, this construct was ineffective in the current trial.

Similar measurement (attenuation, response shifts) and method (e.g., time-frame) artefacts as highlighted above may account for this result. Still, the finding is suggestive that our current psychological constructs may be inadequate to account for behavioral changes. This also reflects correlational research where even our best prediction models typically explain less than 30% of the variance in PA [4]. An expansion of current models to include additional relevant constructs seems prudent yet most of our current theoretical conceptions share considerable redundancy [7,15,26] and thus it is difficult to make suggestions based on the extant literature. Perhaps more sophisticated tests such as an examination of moderators such as personality and environmental characteristics (see Rhodes and Smith [41]) or more specific measurement domains (e.g., belief level vs. aggregation) would be helpful (see Vallance et al. [42]). It is also important to note that the behavior change effect in this study was small (2% variance). While this is typical of worksite interventions [6] and behavioral interventions more generally [5] the precision needed from the purported mediators to account for this very small effect may be difficult. However, it is important that physical activity interventions perform and report mediation analyses even if mediation is not established.

Finally, we must acknowledge the study limitations which include the use of self-report measures to assess physical activity, and the low level of participant retention throughout the 12 month study period. This should be taken into account in the interpretation of our findings.

In summary, an examination of mediators of PA behavior change using four leading social cognition theories was unable to account for the effect of the intervention on behavior in a worksite intervention. The results highlight the importance of reporting mediation results in randomized controlled trials whether the findings are null or positive. Future studies may wish to focus on more detailed dose-response issues between mediators and behavior, the inclusion of moderators that could affect individual change, or different mediator constructs at higher levels of measurement specificity. Overall, however, the results show a similar pattern to prior research where interventions did not result in a change in mediation of behavior. Continued work on innovative and more powerful intervention approaches to PA is timely.

## Competing interests

The authors declare that they have no competing interests.

## Authors' contributions

RCP, MAP, KSC and JCS conceived the study. MAP analyzed the data. MAP, RCP, KSC and JCS interpreted the data. MAP, RCP and RER were responsible for drafting the manuscript. All authors critically evaluated the article for content and approved the final version.
